# Metabolomics reveals the effect of Xuefu Zhuyu Decoction on plasma metabolism in rats with acute traumatic brain injury

**DOI:** 10.18632/oncotarget.21876

**Published:** 2017-10-16

**Authors:** Dandan Feng, Zian Xia, Jing Zhou, Hongmei Lu, Chunhu Zhang, Rong Fan, Xingui Xiong, Hanjin Cui, Pingping Gan, Wei Huang, Weijun Peng, Feng He, Zhiming Wang, Yang Wang, Tao Tang

**Affiliations:** ^1^ Institute of Integrative Chinese Medicine, Xiangya Hospital, Central South University, Changsha 410008, P.R. China; ^2^ Research Center of Modernization of Traditional Chinese Medicines, College of Chemistry and Chemical Engineering, Central South University, Changsha 410083, P.R. China; ^3^ Department of Oncology, Xiangya Hospital, Central South University, Changsha 410008, P.R. China; ^4^ Department of Integrated Chinese and Western Medicine, The Second Xiangya Hospital, Central South University, Changsha 410011, P.R. China; ^5^ Department of Hepatobiliary Surgery, Xiangya Hospital, Central South University, Changsha 410008, P.R. China

**Keywords:** metabolomics, acute traumatic brain injury, traditional Chinese medicine, Xuefu Zhuyu Decoction, gas chromatography/mass spectrometry

## Abstract

Xuefu Zhuyu Decoction (XFZY), an important traditional Chinese herbal formula, has been reported effective on traumatic brain injury (TBI) in rats. However, its cerebral protection mechanism has not been clarified at the metabolic level. This work aims to explore the global metabolic characteristics of XFZY in rats during the acute phase of TBI on days 1 and 3. A plasma metabolomics method based on gas chromatography-mass spectrometry coupled with univariate analysis and multivariate statistical analysis was performed in three groups (Sham, Vehicle, XFZY). Then, a pathway analysis using MetaboAnalyst 3.0 was performed to illustrate the pathways of therapeutic action of XFZY in TBI. XFZY treatment attenuates neurological dysfunction and cortical lesion volume post-injury on day 3, and reverses the plasma metabolite abnormalities (glutamic acid, lactic acid, 3-hydroxybutyric acid, and ribitol, etc.). These differential metabolites are mainly involved in D-glutamine and D-glutamate metabolism, alanine, aspartate and glutamate metabolism, and inositol phosphate metabolism. Our study reveals potential biomarkers and metabolic networks of acute TBI and neuroprotection effects of XFZY, and shows this metabolomics approach with MetaboAnalyst would be a feasible way to systematically study therapeutic effects of XFZY on TBI.

## INTRODUCTION

Traumatic brain injury (TBI) is the major cause of death and disability in individuals under the age of 45 years [1, 2]. It can lead to temporary or permanent impairment of cognitive, physical and psychosocial functions [3], and thus brings a significant social and economic burden. However, it is frequently referred to as the “silent epidemic” because of undetected complications that resulted from TBI and limited awareness, funding, and research progress [4, 5]. In the United State, an estimated 1.7 million people suffer from TBI annually [6], and about 5.3 million people live with a TBI-related disability [7]. Despite progress made in diagnosis, neurological care, and functional rehabilitation, no effective therapy is currently available for TBI [8]. Since TBI is not a single pathophysiological event but a complex disease process [9], it is urgent to seek a comprehensive treatment for TBI.

Fortunately, traditional Chinese medicine (TCM) formula is an herb combination and contains multiple components that could impact multiple targets and induce synergistic therapeutic efficacies [10]. Furthermore, an evidence-based review comprising 25 studies concluded that 15 TCM formula, such as modified “Shengyu” decoction, Huayu capsules, Quyu Tongfu decoction, Angong Niuhuang pill, and Longxuejie capsules, potentially showed neuroprotective effects in terms of reducing brain water content, improving blood-brain barrier permeability, and decreasing TNF-α/NO expression in animals after TBI [11]. Therefore, we considered that TCM formula could be potentially used to develop an effective treatment for TBI.

Xuefu Zhuyu Decoction (XFZY), a TCM herbal formula, has been used to treat several cardio-cerebral diseases including hypertension [12], hyperlipemia [13], thromboembolic stroke [14], ischemic stroke [15], and traumatic brain injury [16]. It is recorded in the book “Yi Lin Gai Cuo” (Correction on Errors in Medical Classics) by Qingren Wang in the Qing Dynasty, for its efficacy in promoting the blood circulation to remove blood stasis and activating the flow of Qi to relieve pain. It consists of the following 11 herbs (ratio is shown in Table 1): Semen Persicae, Flos Carthami, Radix Angelicae Sinensis, Radix Rehmanniae, Radix Achyranthis Bidentatae, Radix Paeoniae Rubra, Fructus Aurantii, Rhizoma Chuanxiong, Radix Platycodi, Radix Bupleuri, Radix Glycyrrhizae. Our previous study [16] demonstrated that XFZY exerts neuroprotection effects and reduces inflammation post-TBI in rats via a mechanism involving downregulation of AKT/mTOR/p70S6K proteins in brain tissues, especially at a dose of 9 g/kg. However, little is known about changes in overall plasma metabolites upon XFZY administration in a rat model of TBI.

**Table 1:** Components of Xuefu Zhuyu Decoction

Chinese Name	Latin Name	Plant Name	Ratio	Source	Specimen number
Tao Ren	Semen *Persicae*	*Prunus persica* (L.) Batsch	8	Sichuan	201305170
Hong Hua	Flos *Carthami*	*Carthamus tinctorius* L.	6	Henan	201304182
Dang Gui	Radix *Angelicae Sinensis*	*Angelica sinensis* (Oliv.) Diels	6	Gansu	201303210
Sheng Di	Radix *Rehmanniae*	*Rehmannia glutinosa* Libosch.	6	Henan	201303151
Niu Xi	Radix *Achyranthis Bidentatae*	*Achyranthes bidentata* Bl.	6	Sichuan	201303232
Chi Shao	Radix *Paeoniae Rubra*	*Paeonia lactiflora* Pall.	4	Anhui	201304163
Zhi Qiao	Fructus *Aurantii*	*Citrus aurantium* L.	4	Hunan	201304131
Gan Cao	Radix *Glycyrrhizae*	*Glycyrrhiza uralensis* Fisch.	4	Ningxia	201306190
Chuan Xiong	Rhizoma *Chuanxiong*	*Ligusticumi chuanxiong* Hort.	3	Sichuan	201305191
Jie Geng	Radix *Platycodonis*	*Platycodon grandiflorum* (Jacq.) A. DC.	3	Hebei	201211032
Chai Hu	Radix *Bupleuri*	*Bupleurum chinense* DC.	2	Hebei	201301310

A systems perspective regarding effects of XFZY on TBI is desirable, where details of various system components can be integrated with increasing complexity to better understand properties of the entire system [17]. As an omics science in systems biology, metabolomics, focusing on the global metabolite profiles in various biological samples such as urine, plasma or tissues, have been widely used to explore metabolic abnormalities in TBI [18, 19] and metabolic effects of TCM formula [20, 21]. Metabolomics provides a new vision of the holistic effects and synergistic efficacy of TCM formula [13]. High sensitivity and numerous available databases make gas chromatography-mass spectrometry (GC-MS) a powerful tool for use in metabolomics studies [22]. The metabolic response of XFZY to TBI induced by Feeney’s weight-dropping method has been demonstrated by nuclear magnetic resonance (NMR)-based metabolomics [23], which was only reported in Chinese. Therefore, a GC-MS based plasma metabolomics study could be carried out to observe the overall metabolites changes upon XFZY treatment in rats after TBI.

**Figure 1:**
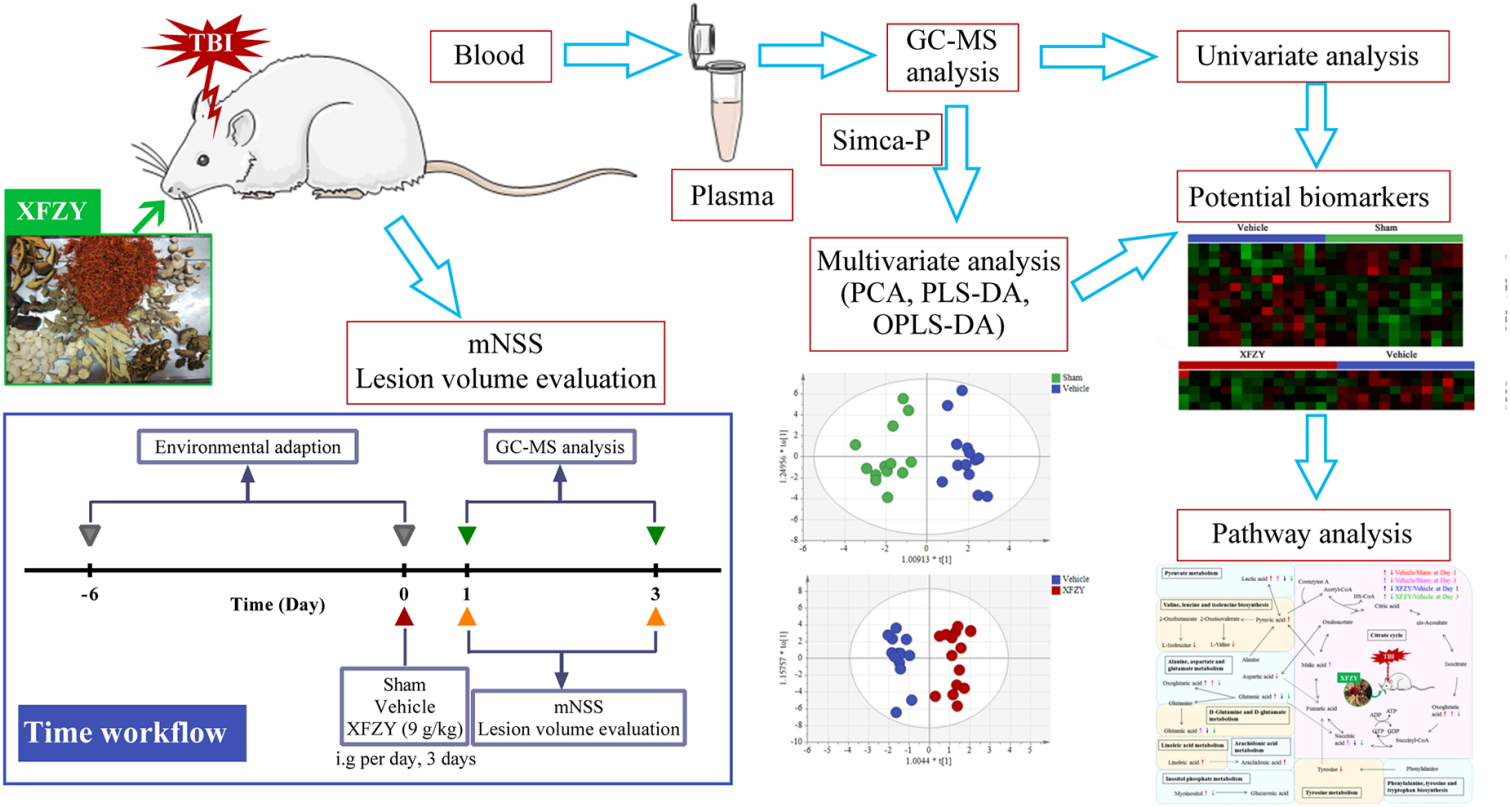
Flow chart of the present study A controlled cortical impact injury (CCI)-induced TBI model in rats was established, and then rats were treated with 9 g/kg XFZY for three consecutive days. The modified neurologic severity score and lesion volume was evaluated to assess neurological deficits of TBI and effects of XFZY. Then a gas chromatography-mass spectrometer (GC-MS)-based plasma metabolomics coupled with univariate and multivariate statistical analysis was used to identify potential biomarkers. Finally, the pathway analysis was performed to illustrate pathways of therapeutic action of XFZY in TBI.

In this work, we applied a GC-MS based plasma metabolomics approach coupled with pattern recognition and a pathway analysis to monitor metabolic changes of controlled cortical impact injury (CCI)-induced TBI during acute phases on day 1 and day 3 and the response to 9 g/kg XFZY treatment. Furthermore, we illustrated a network of canonical metabolic pathways to explain the biochemical mechanism, and thus, provide a multiple targets explanation on effects of XFZY on TBI. The workflow is illustrated in Figure 1.

## RESULTS

During this study, because of anesthesia, operation, or intragastrical gavage, two rats died in Sham and Vehicle group respectively. The mortality was 13.3 % (2/15) in the Sham group, 7.4 % (2/27) in the Vehicle group, and 0 (0/27) in the XFZY group. There are no differences of mortality among the three groups (*p* > 0.05). Four rats that died were excluded from the final data analysis. Furthermore, XFZY could slightly, not significantly, alleviate the decrease of body weight in rats post-CCI on day 1 and accelerate the increase of body weight on day 3 (data not shown).

### XFZY significantly decreases modified neurologic severity score of rats after CCI on day 3

**Figure 2:**
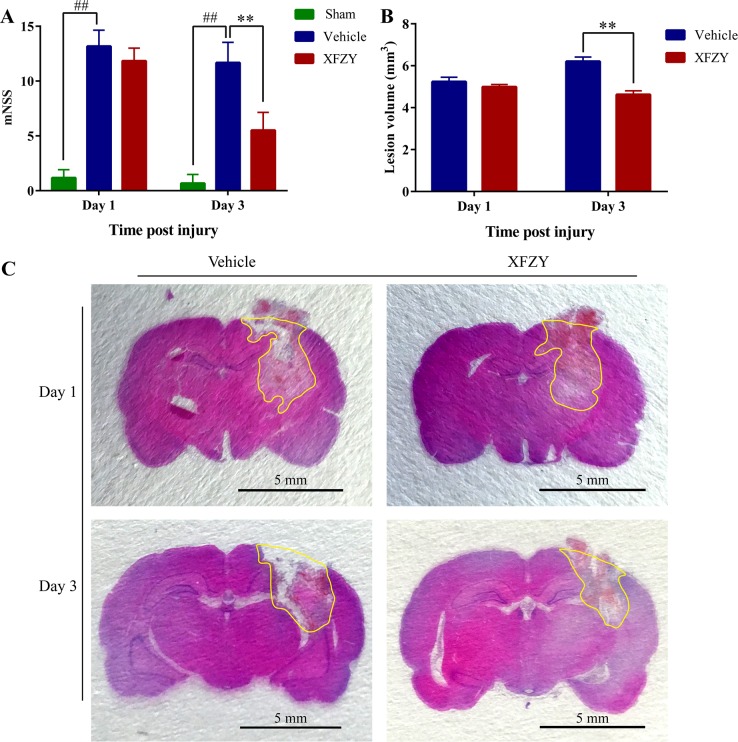
Effects of XFZY on modified neurologic severity score and lesion volume in rats post-CCI on day 1 and day 3 **(A)** Rats (n=6/group) subjected to CCI exhibit neurologic deficits from days 1 to 3. Maximal neurologic deficit score is 18; normal score is 0. XFZY group has differently lower scores than the Vehicle group on day 3 post-CCI. **(B)** Measurement of brain lesion volume by HE staining in Vehicle and XFZY groups. XFZY treatment on day 3 significantly reduces lesion volume when compared to saline treatment. **(C)** Representative photographs of 3-μm-thick coronal slices from each group with H&E staining are shown. Lesion regions are circled by yellow lines (n=6/group, scale bar, 5.0 mm, values are presented as the mean ± SD, ^#^*p* < 0.05, ^##^*p* < 0.01 *vs*. Sham group. ^*^p < 0.05, ^**^p < 0.01 *vs*. Vehicle group).

We performed the modified Neurological Severity Score (mNSS) test to evaluate the neurological outcome of TBI rats. A lower score in the mNSS test demonstrates a better neurological function. As shown in Figure 2A (n=6/group), TBI dramatically impairs neurological function on day 1 (*p* < 0.01) and day 3 (*p* < 0.01). Treatment with XFZY (9 g/kg) considerably ameliorates neurological dysfunction after TBI on day 3 (*p* < 0.01), whereas no significant difference is observed on day 1 (*p* = 0.36), which is similar to our previous study [16].

### XFZY treatment notedly decreases CCI-induced lesion volume on day 3

XFZY treatment on day 3 results in a significant reduction in lesion size after injury as compared with Vehicle group (*p* < 0.01, n = 6/group, Figure 2B, 2C), whereas with a nonsignificant reduction on day 1. The present study demonstrates that post-injury administration of XFZY produces a significant attenuation of brain lesion volume associated with TBI in rats on day 3.

### The OPLS-DA model shows XFZY can effectively reverse the abnormal metabolic profile of rats post-CCI on day 1, 3

**Figure 3:**
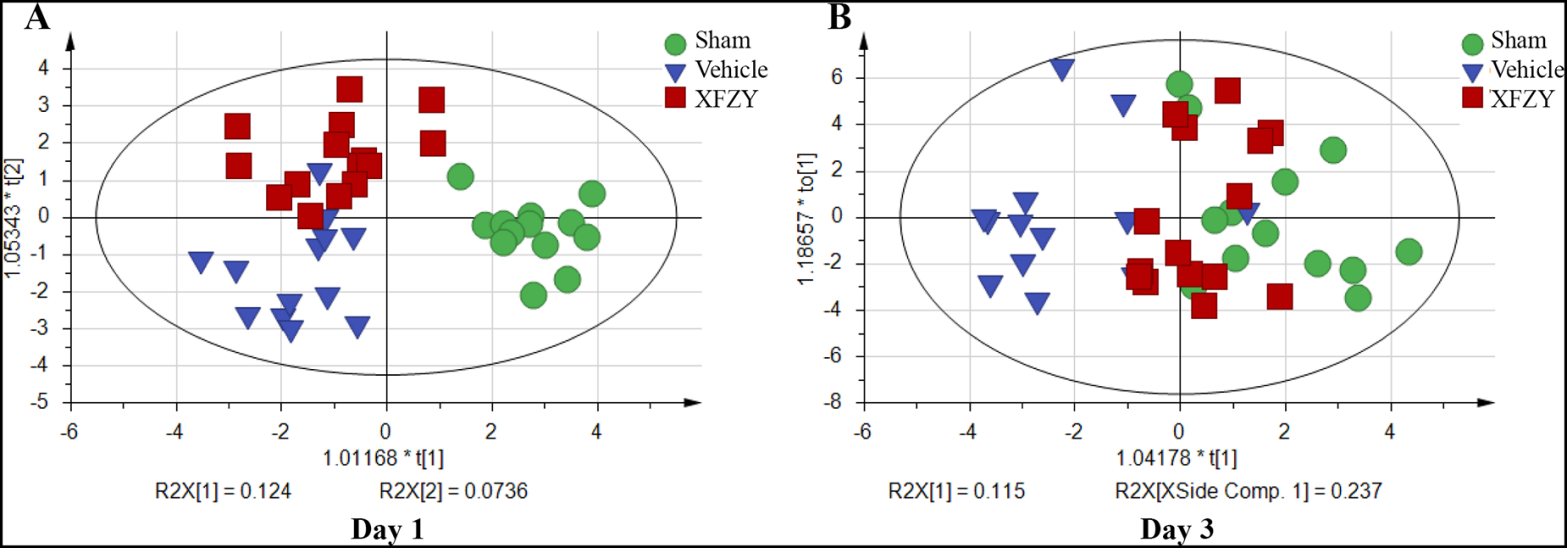
Effects of XFZY on abnormal metabolic profile of rats post-CCI on day 1, 3 OPLS-DA score plot of Sham (green circle), Vehicle (dark blue triangle), XFZY (dark red square) groups on day 1 **(A)** and day 3 **(B)** post-injury. XFZY can effectively reverse the abnormal metabolic profile of rats post-CCI on day 1, 3.

As shown in Table 2, we qualitatively and quantitatively identified 37 plasma metabolites via a GC-MS-based metabolomics approach and then performed a multivariate analysis to determine the metabolites in these three groups. A summary of parameters for assessing the quality of multivariate analysis is shown in Table 3. Figure 3 showed that the metabolic profile of XFZY treatment group is closer to the Sham group than the Vehicle group on day 1 (Figure 3A) and day 3 (Figure 3B), in addition that on day 3 is much closer than on day 1. It indicated that XFZY can effectively reverse the abnormal metabolic profile of rats post-CCI.

**Table 2:** Qualitative and quantitative analysis of metabolic profiles of Sham, Vehicle and XFZY rats on day 1 and day 3

No.	Metabolites	Formula	Actual mass	Relative concentrations (mg/ml)						
					day 1			day 3		
				RSD of QC	Sham (n=13)	Vehicle (n=13)	XFZY (n=15)	Sham (n=13)	Vehicle (n=13)	XFZY (n=15)
1	Pyruvic acid	C_3_H_4_O_3_	88.0621	12.927%	0.065±0.014	0.085±0.020	0.082±0.010	0.113±0.022	0.123±0.030	0.118±0.034
2	L-Lactic acid^a^	C_3_H_6_O_3_	90.0779	5.213%	4.361±0.370	5.077±0.895	4.341±0.507	3.088±0.647	4.278±0.477	3.310±0.508
3	L-Alanine^a^	C_3_H_7_NO_2_	89.0932	12.375%	0.473±0.061	0.426±0.083	0.415±0.065	0.386±0.126	0.431±0.181	0.422±0.126
4	Glycine^a^	C_2_H_5_NO_2_	75.0666	20.651%	0.249±0.031	0.362±0.320	0.244±0.050	0.266±0.096	0.245±0.096	0.265±0.062
5	Methylmalonic acid	C_4_H_6_O_4_	118.088	15.462%	0.058±0.008	0.061±0.012	0.054±0.008	0.064±0.010	0.067±0.013	0.064±0.016
6	Acetylglycine	C_4_H_7_NO_3_	117.1033	12.008%	0.076±0.008	0.077±0.011	0.074±0.009	0.075±0.009	0.079±0.010	0.077±0.008
7	3-Hydroxybutyric acid^a^	C_4_H_8_O_3_	104.1045	5.139%	0.074±0.015	0.187±0.179	0.085±0.041	0.296±0.214	0.797±0.457	0.645±0.540
8	L-Valine^a^	C_5_H_11_NO_2_	117.1463	14.423%	0.213±0.027	0.171±0.037	0.171±0.044	0.165±0.049	0.177±0.055	0.170±0.038
9	Urea	CH_4_N_2_O	60.0553	17.115%	2.849±0.638	2.980±0.674	3.189±0.404	2.830±0.813	2.802±1.063	2.855±0.670
10	L-Isoleucine^a^	C_6_H_13_NO_2_	131.1729	11.970%	0.267±0.034	0.233±0.046	0.221±0.028	0.235±0.055	0.239±0.051	0.224±0.033
11	L-Proline^a^	C_5_H_9_NO_2_	115.1305	16.876%	0.323±0.049	0.213±0.062	0.227±0.051	0.210±0.124	0.186±0.092	0.208±0.084
12	Succinic acid	C_4_H_6_O_4_	118.088	13.076%	0.024±0.005	0.027±0.005	0.022±0.004	0.019±0.006	0.028±0.006	0.023±0.009
13	Glyceric acid	C_3_H_6_O_4_	106.0773	18.739%	0.039±0.015	0.045±0.015	0.046±0.015	0.027±0.015	0.031±0.007	0.027±0.012
14	L-Serine^a^	C_3_H_7_NO_3_	105.0926	9.985%	0.296±0.042	0.287±0.054	0.258±0.036	0.281±0.070	0.264±0.069	0.269±0.033
15	L-Threonine^a^	C_4_H_9_NO_3_	119.1192	8.712%	0.423±0.055	0.385±0.057	0.371±0.070	0.397±0.091	0.322±0.088	0.346±0.056
16	L-Malic acid^a^	C_4_H_6_O_5_	134.0874	10.501%	0.027±0.007	0.029±0.011	0.026±0.006	0.029±0.005	0.038±0.010	0.035±0.009
17	Pyroglutamic acid^a^	C_5_H_7_NO_3_	129.114	13.483%	0.492±0.188	0.594±0.102	0.535±0.092	0.599±0.088	0.648±0.121	0.577±0.095
18	4-Hydroxyproline	C_5_H_9_NO_3_	131.1299	9.072%	0.092±0.014	0.093±0.020	0.093±0.014	0.101±0.020	0.085±0.022	0.090±0.018
19	Creatinine enol	C_4_H_7_N_3_O	113.1179	15.175%	0.104±0.017	0.144±0.035	0.125±0.042	0.119±0.039	0.158±0.052	0.147±0.039
20	Oxoglutaric acid	C_5_H_6_O_5_	146.0981	28.139%	0.009±0.003	0.011±0.003	0.010±0.005	0.009±0.003	0.014±0.005	0.011±0.003
21	L-Glutamic acid^a^	C_5_H_9_NO_4_	147.1293	10.694%	0.079±0.017	0.095±0.029	0.073±0.013	0.072±0.021	0.089±0.017	0.071±0.011
22	L-Phenylalanine^a^	C_9_H_11_NO_2_	165.1891	8.115%	0.084±0.014	0.078±0.011	0.072±0.010	0.074±0.015	0.080±0.015	0.075±0.012
23	L-Aspartic acid	C_4_H_7_NO_4_	133.1027	16.927%	0.031±0.013	0.032±0.020	0.026±0.007	0.030±0.009	0.024±0.006	0.030±0.016
24	Ribitol	C_5_H_12_O_5_	152.1458	10.406%	0.059±0.018	0.081±0.020	0.094±0.009	0.057±0.012	0.063±0.015	0.058±0.016
25	Citric acid^a^	C_6_H_8_O_7_	192.1235	26.040%	0.071±0.022	0.065±0.024	0.061±0.027	0.081±0.024	0.088±0.026	0.082±0.028
26	1,5-Anhydrosorbitol	C_6_H_12_O_5_	164.1565	11.321%	0.129±0.037	0.124±0.025	0.136±0.025	0.137±0.025	0.135±0.020	0.133±0.017
27	D-Glucose^a^	C_6_H_12_O_6_	180.1559	14.060%	7.449±1.494	7.077±1.577	7.456±0.978	8.300±1.156	7.460±1.355	7.416±1.088
28	L-Tyrosine^a^	C_9_H_11_NO_3_	181.1885	16.849%	0.092±0.023	0.074±0.016	0.078±0.021	0.078±0.021	0.071±0.016	0.077±0.017
29	Palmitic acid^a^	C_16_H_32_O_2_	256.4241	19.500%	0.479±0.090	0.584±0.168	0.564±0.120	0.533±0.128	0.519±0.188	0.541±0.171
30	Myo-inositol	C_6_H_12_O_6_	180.1559	14.866%	0.132±0.021	0.153±0.034	0.152±0.046	0.118±0.027	0.153±0.036	0.125±0.017
31	Heptadecanoic acid	C_17_H_34_O_2_	270.4507	24.965%	0.011±0.002	0.013±0.004	0.010±0.002	0.010±0.003	0.010±0.002	0.010±0.003
32	Linoleic acid^a^	C_18_H_32_O_2_	280.4455	13.746%	0.299±0.053	0.370±0.104	0.357±0.079	0.377±0.099	0.435±0.091	0.384±0.125
33	Oleic acid^a^	C_18_H_34_O_2_	282.4614	10.289%	0.286±0.043	0.345±0.096	0.359±0.052	0.346±0.083	0.411±0.107	0.380±0.133
34	Stearic acid^a^	C_18_H_36_O_2_	284.4772	26.825%	0.285±0.073	0.310±0.063	0.292±0.056	0.290±0.071	0.276±0.042	0.288±0.067
35	Arachidonic acid^a^	C_20_H_32_O_2_	304.4669	14.157%	0.075±0.018	0.105±0.024	0.089±0.021	0.111±0.036	0.104±0.033	0.103±0.032
36	Cholesterol^a^	C_27_H_46_O	386.6535	15.215%	0.318±0.067	0.411±0.160	0.402±0.103	0.442±0.094	0.460±0.127	0.411±0.093
37	Phosphate	O_4_P	94.9714	9.022%	0.678±0.131	0.865±0.195	0.787±0.116	0.738±0.194	0.816±0.233	0.752±0.230

Concentration of 37 metabolites are presented as the mean ± SD by the ratio of its peaks area to that of the internal standard on the same chromatogram.

^a^ Identified by standard substances.

**Table 3:** Summary of parameters for assessing the quality of PCA and OPLS-DA models

Samples	Models	No.^a^	R^2^X_cum_^b^	R^2^Y_cum_^b^	Q^2^Y_cum_^b^	R intercept ^c^	Q intercept ^c^
Day 1							
Three groups	PCA	4	0.571	-	0.128	-	-
Three groups	OPLS-DA	2+2	0.513	0.718	0.399	-	-
Vehicle *vs.* Sham	PLS-DA	3	0.504	0.918	0.737	0.599	-0.407
XFZY *vs.* Vehicle	PLS-DA	2	0.356	0.609	0.0941	0.526	-0.166
Vehicle *vs.* Sham	OPLS-DA	1+2	0.504	0.918	0.761	-	-
XFZY *vs.* Vehicle	OPLS-DA	1+1	0.356	0.609	0.107	-	-
Day 3							
Three groups	PCA	4	0.619	-	0.24	-	-
Three groups	OPLS-DA	1+1	0.325	0.314	0.109	-	-
Vehicle *vs.* Sham	PLS-DA	2	0.349	0.764	0.377	0.491	-0.239
XFZY *vs.* Vehicle	PLS-DA	2	0.357	0.671	0.28	0.475	-0.204
Vehicle *vs.* Sham	OPLS-DA	1+3	0.574	0.904	0.492	-	-
XFZY *vs.* Vehicle	OPLS-DA	1+4	0.601	0.921	0.411	-	-

^a^ No. is the number of components.^b^ R^2^X_cum_ and R^2^Y_cum_ are the cumulative modeled variations in the X and Y matrices, respectively, and Q^2^Y_cum_ is the cumulative predicted variation in Y matrix. ^c^ R and Q were obtained after PLS-DA permutation tests (n=200).

**Figure 4:**
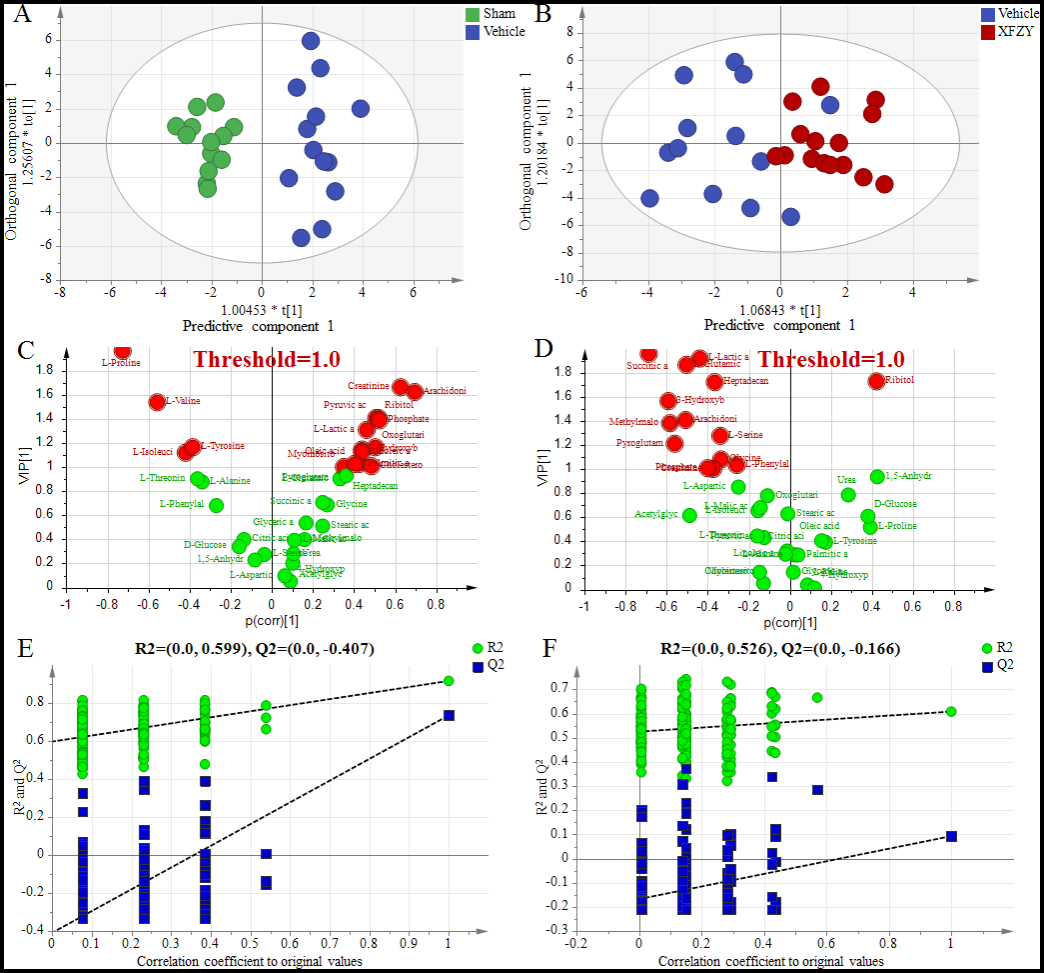
Differentiation of the Sham, Vehicle and XFZY groups on day 1 using multivariate analysis based on plasma spectral data of GC-MS **(A)** OPLS-DA score plot of the Vehicle group (dark blue) and the Sham group (green) on day 1 (1+2 components, R2X=0.504, R2Y=0.918, Q2Y=0.761). **(B)** OPLS-DA score plot of the Vehicle group (dark blue) and XFZY group (dark red) on the day 1 (1+1 components, R2X=0.356, R2Y=0.609, Q2Y=0.107). t [1] = scores for predictive component 1, to [1] = scores for orthogonal component 1. The ellipse shows the 95% confidence interval using Hotelling T^2^ statistics. **(C** and **D)** VIP value plots from OPLS-DA based on plasma profiling of the Vehicle *vs*. Sham group and XFZY *vs*. Vehicle group on day 1, respectively. Metabolites with VIP >1.0 are highlighted with red. Metabolites with p(corr)>0 represent metabolites increase in the Vehicle group *vs*. the Sham or in the XFZY group *vs*. the Vehicle group, metabolites with p(corr)<0 represent a decrease. **(E** and **F)** Validation plots of the partial least squares discriminant analysis (PLS-DA) models acquired through 200 permutation tests for the Vehicle *vs*. Sham groups and XFZY *vs*. Vehicle groups, respectively. R2 (green circles) and Q2 (blue boxes) values from the permuted analysis (bottom left) are significantly lower than the corresponding original R2 and Q2 values (top right), suggesting that the constructed PLS-DA model is valid and not overfitted.

**Figure 5:**
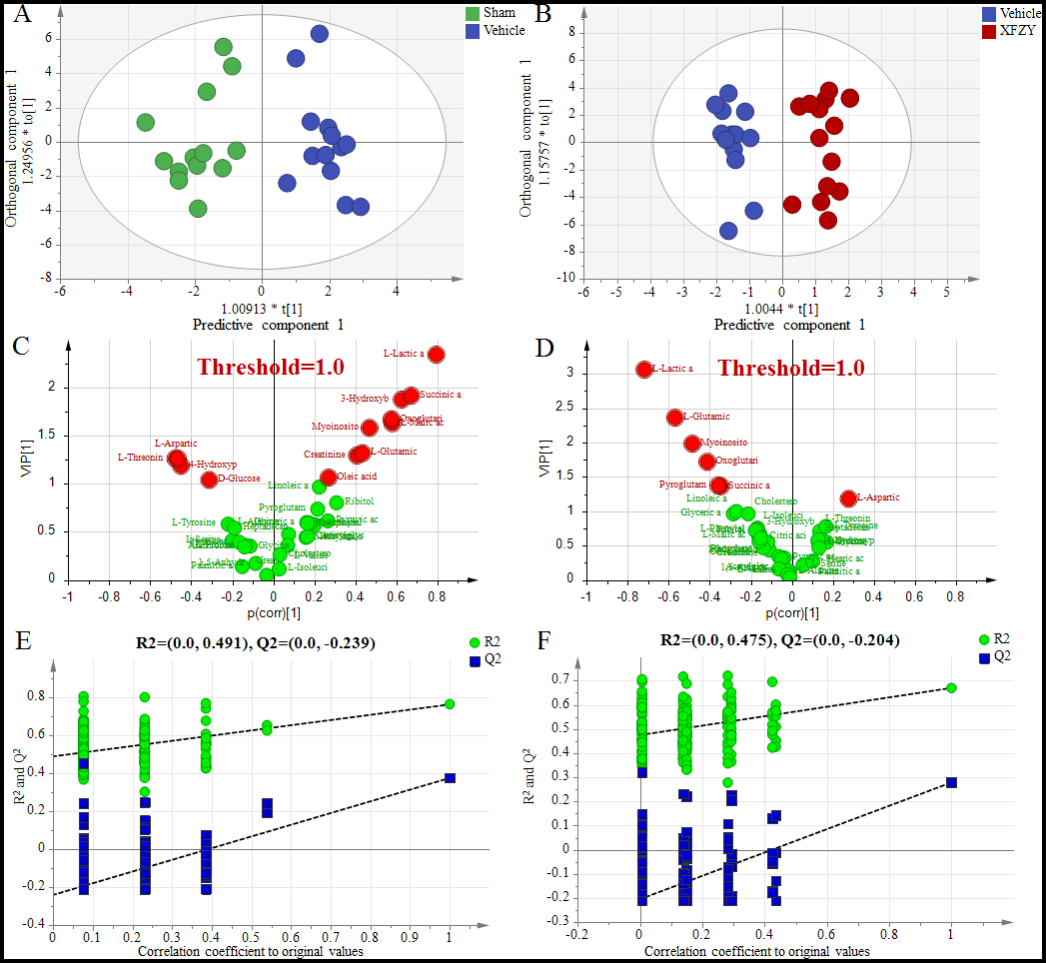
Differentiation of the Sham, Vehicle and XFZY groups on day 3 using multivariate analysis based on plasma spectral data of GC-MS **(A)** OPLS-DA score plot for the Vehicle group (dark blue) and Sham group (green) on day 3 (1+3 components, R2X=0.574, R2Y=0.904, Q2Y=0.492). **(B)** OPLS-DA score plot for the Vehicle group (dark blue) and XFZY group (dark red) on the day 3 (1+4 components, R2X=0.601, R2Y=0.921, Q2Y=0.411). t [1] = scores for predictive component 1, to [1] = scores for orthogonal component 1. The ellipse shows the 95% confidence interval using Hotelling T^2^ statistics. **(C** and **D)** VIP value plots from OPLS-DA based on plasma profiling of the Vehicle *vs.* Sham group and XFZY *vs.* Vehicle group on day 3, respectively. Metabolites with VIP >1.0 are highlighted with red. Metabolites with p(corr)>0 represent metabolites increase in the Vehicle group *vs*. the Sham group or in the XFZY group *vs*. the Vehicle group, while metabolites with p(corr)<0 represent a decrease. **(E** and **F)** Validation plots of the partial least squares discriminant analysis (PLS-DA) models acquired through 200 permutation tests for the Vehicle *vs.* Sham groups and the XFZY *vs.* Vehicle groups, respectively. R2 (green circles) and Q2 (blue boxes) values from the permuted analysis (bottom left) are significantly lower than the corresponding original R2 and Q2 values (top right), suggesting that the constructed PLS-DA model is valid and not overfitted.

For visualizing the different metabolic profiling among Vehicle group *vs*. Sham, or XFZY groups, principal component analysis (PCA) was firstly used to analyze the expression levels of metabolic biomarkers in different groups. PCA score plots show a trend of separation among the three groups on day 1 and day 3 (Supplementary Figure 1A, 1B). Then partial least-square discrimination analysis (PLS-DA) and orthogonal partial least-squares discriminant analysis (OPLS-DA) were used to maximize the plasma metabolite pattern among the Vehicle *vs.* Sham group and XFZY *vs.* Vehicle group. The score plots of plasma spectral data based on the PLS-DA and OPLS-DA model show a clear separation without any overlap between the Vehicle and Sham groups both on day 1 and day 3 (Figures 4A, 5A, Supplementary Figure 1C, 1D), implying that the metabolic profile significantly altered in rats post-CCI.

PLS-DA score plots present a separation trend with an overlap between the Vehicle and XFZY groups on day 1 and day 3 (Supplementary Figure 1E, 1F). However, OPLS-DA score plots of plasma spectral data show an obvious separation without any overlap between the TBI and XFZY groups on day 3 (Figure 5B) with class discrimination statistical parameters R2Y 0.921 and Q2 0.411. Whereas a separation tendency is found on day 1 with R2Y 0.609 and Q2 0.107 (Figure 4B), implying that the metabolic profile significantly altered in XFZY-treated rats with TBI. These parameters of the data indicated that the models are of good quality and provide accurate predictions.

To validate PLS-DA models, 200 random permutation tests were performed to produce intercepts for all the data. As shown in Figure 4E, 4F, and Figure 5E, 5F, validation plots show R2 (green circles) and Q2 (blue boxes) values from the permuted analysis (bottom left) are significantly lower than the corresponding original R2 and Q2 values (top right). All of the validation plots indicated that all the constructed PLS-DA models are valid and without overfitting.

### Metabolite abnormalities of the Vehicle group *vs*. Sham and XFZY groups on day 1 and day 3

**Figure 6:**
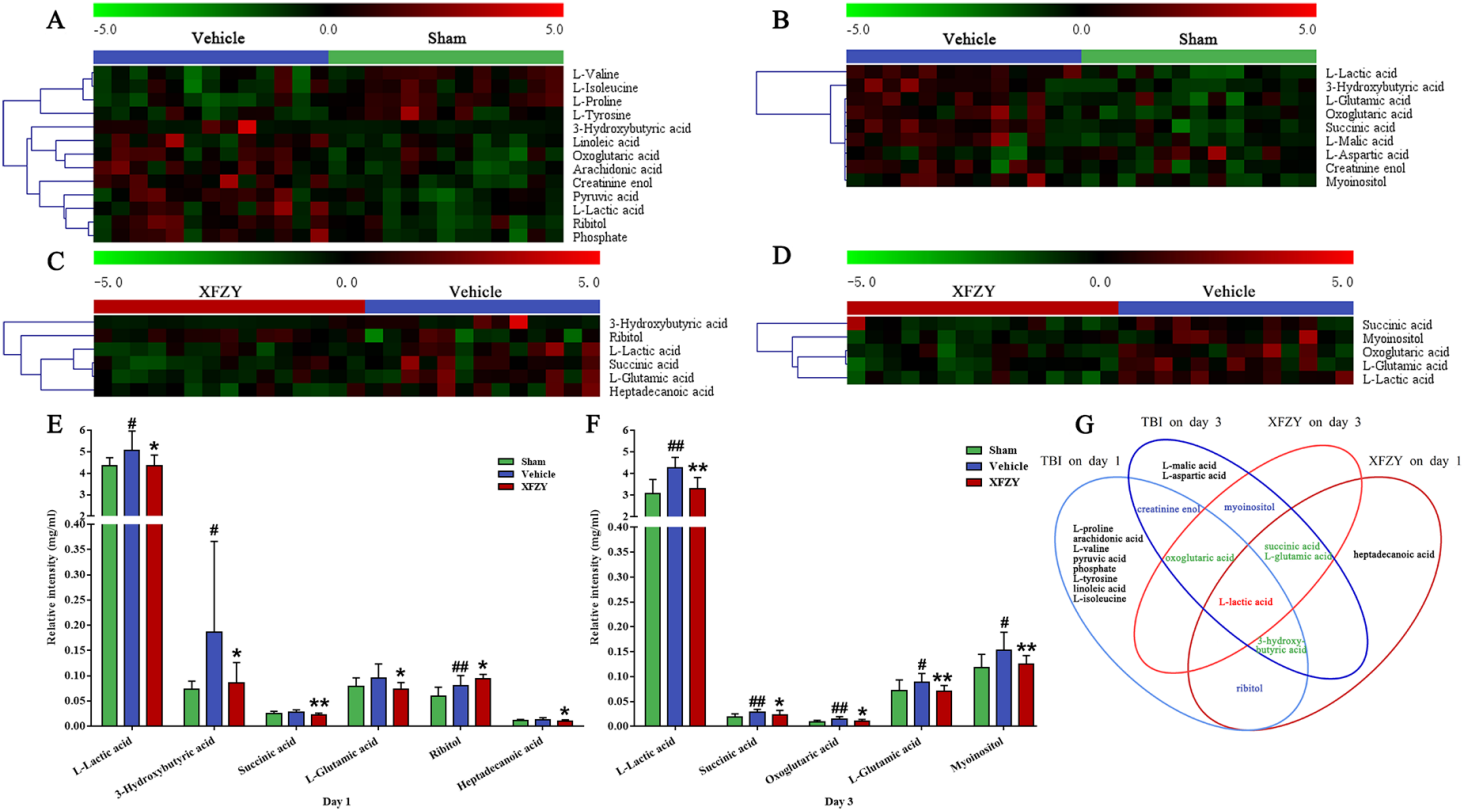
Significant metabolites identified on day 1 and day 3 Heat maps of the significant metabolites in plasma between the Vehicle and Sham groups on day 1 **(A)** and day 3 **(B)**. Heat maps of the significant metabolites in plasma between the XFZY and Vehicle groups on day 1 **(C)** and day 3 **(D)**. (Rows: relative concentrations of significant metabolites after mean centering and unit variance scaling of the data; Columns: plasma samples from different groups; color scale indicates metabolite expression value, green is lowest and red is highest.) **(E)** Quantification of significant metabolites associated with the treatment of XFZY in the three groups on day 1. **(F)** Quantification of significant metabolites associated with the treatment of XFZY in the three groups on day 3 (Green: Sham; dark blue: Vehicle; dark red: XFZY. mean ± SD, ^#^*p* < 0.05, ^##^*p* < 0.01 *vs*. the Sham group; ^*^p < 0.05, ^**^p < 0.01 *vs*. the Vehicle group, Student’s t-tests with Welch’s correction or Mann-Whitney U-tests). **(G)** Venn diagram of the significant metabolites associated with TBI and the treatment of XFZY on day 1 and day 3.

The vehicle group could be separated completely from the Sham and XFZY group in the OPLS-DA score plots on day 1 and day 3, which indicated that plasma metabolism significantly changed in acute TBI and XFZY-treated rats. In the present work, value of variable importance in the projection (VIP) above 1.0 is used as a screening standard to select potential metabolites, marked by red in the VIP value plots (Figure 4C, 4D, Figure 5C, 5D). Metabolites in both terminals of V represent a high contribution to the discrimination of the group separation. Compared to the Sham group (Vehicle group), increased metabolites in the Vehicle group (XFZY group) are in the right quadrant (positive covariance) and decreased metabolites are in the left quadrant (negative covariance). In addition, metabolites with VIP values >1.0 and *p*-values < 0.05 in every group are deemed to be statistically significant, which are shown in Table 4 and Figure 6E-6G. To visually display the variation of concentrations of significant metabolites between Vehicle group *vs*. Sham or XFZY groups on day 1 and day 3, heat maps were produced according to the relative quantities of each metabolite by the Pearson correlation and the average linkage method (Figure 6A-6D). The higher concentration of metabolites, the redder in the heat map, and the lower concentration of metabolites, the greener in the heat map.

**Table 4:** List of significant metabolites in different groups identified from the OPLS-DA model

			Vehicle *vs.* Sham on day 1			XFZY *vs.* Vehicle on day 1			Vehicle *vs.* Sham on day 3			XFZY *vs.* Vehicle on day 3		
Metabolites	HMDB	KEGG	VIP^a^	FC^b^	*p*-value^c^	VIP^a^	FC^b^	*p*-value^c^	VIP^a^	FC^b^	*p*-value^c^	VIP^a^	FC^b^	*p*-value^c^
L-Proline	HMDB00162	C00148	1.97	↓0.66	0.000	-	-	-	-	-	-	-	-	-
Creatinine enol	HMDB00562	C00791	1.67	↑1.39	0.002	-	-	-	1.292	↑1.333	0.039	-	-	-
Arachidonic acid	HMDB01043	C00219	1.63	↑1.40	0.001	-	-	-	-	-	-	-	-	-
L-Valine	HMDB00883	C00183	1.54	↓0.80	0.003	-	-	-	-	-	-	-	-	-
Pyruvic acid	HMDB00243	C00022	1.41	↑1.30	0.007	-	-	-	-	-	-	-	-	-
Ribitol	HMDB00508	C00474	1.41	↑1.37	0.007	1.73	↑1.17	0.037	-	-	-	-	-	-
Phosphate	HMDB01429	C00009	1.39	↑1.28	0.008	-	-	-	-	-	-	-	-	-
L-Lactic acid	HMDB00190	C00186	1.31	↑1.16	0.017	1.92	↓0.86	0.011	2.34	↑1.39	0.000	2.617	↓0.774	0.000
L-Tyrosine	HMDB00158	C00082	1.17	↓0.81	0.031	-	-	-	-	-	-	-	-	-
Oxoglutaric acid	HMDB00208	C00026	1.16	↑1.28	0.031	-	-	-	1.67	↑1.59	0.006	1.358	↓0.739	0.035
3-Hydroxybutyric acid	HMDB00357	C01089	1.15	↑2.52	0.022^d^	1.57	↓0.46	0.022^d^	1.87	↑2.69	0.002	-	-	-
Linoleic acid	HMDB00673	C01595	1.13	↑1.24	0.041	-	-	-	-	-	-	-	-	-
L-Isoleucine	HMDB00172	C00407	1.12	↓0.87	0.038	-	-	-	-	-	-	-	-	-
Succinic acid	HMDB00254	C00042	-	-	-	1.96	↓0.83	0.010	1.92	↑1.46	0.001	1.084	↓0.818	0.011^d^
L-Glutamic acid	HMDB00148	C00025	-	-	-	1.87	↓0.77	0.023	1.32	↑1.23	0.035	1.862	↓0.798	0.003
Heptadecanoic acid	HMDB02259	-	-	-	-	1.72	↓0.79	0.025	-	-	-	-	-	-
L-Malic acid	HMDB00156	C00149	-	-	-	-	-	-	1.63	↑1.32	0.007	-	-	-
Myo-inositol	HMDB00211	C00137	-	-	-	-	-	-	1.58	↑1.30	0.010	1.586	↓0.817	0.022
L-Aspartic acid	HMDB00191	C00049	-	-	-	-	-	-	1.27	↓0.79	0.044	-	-	-

^a^ Variable importance in the projection (VIP) was obtained based on OPLS-DA with a threshold of 1.0. ^b^ Fold change was calculated based on the arithmetic mean concentration of each group. Fold change with a value more than 1 and “↑” indicates a higher level in the Vehicle group compared to Sham or a higher level in the XFZY group compared to Vehicle, while a value less than 1 and “↓” indicates a lower level in the Vehicle group compared to Sham or a lower level in the XFZY group compared to Vehicle. ^c^ *p*-values calculated using two-tailed Student’s t-tests. ^d^ *p*-values calculated using Mann–Whitney U tests. The short dash line (-) indicates no significant variation.

On day 1 when compared to the Sham group, levels of creatinine enol, arachidonic acid, pyruvic acid, ribitol, phosphate, L-lactic acid, oxoglutaric acid, 3-hydroxybutyric acid, linoleic acid are differently higher, while levels of L-proline, L-valine, L-tyrosine and L-isoleucine are lower in Vehicle group. Then compared XFZY group with the Vehicle group, the level of ribitol significantly increases, while others (L-lactic acid, 3-hydroxybutyric acid, succinic acid, L-glutamic acid and heptadecanoic acid) significantly decrease in plasma samples of the XFZY group.

On day 3 when compared to the Sham group, the level of L-aspartic acid significantly decreases, while others (L-lactic acid, succinic acid, 3-hydroxybutyric acid, oxoglutaric acid, L-malic acid, myo-inositol, L-glutamic acid, and creatinine enol) increase in the Vehicle group. On day 3 when compared with the Vehicle group, levels of L-lactic acid, succinic acid, oxoglutaric acid, myo-inositol, and L-glutamic acid are significantly higher in the XFZY group, which indicated that XFZY might exert its therapeutic effects on TBI through various targets.

### Metabolic pathway analysis of XFZY on rats with TBI

**Figure 7:**
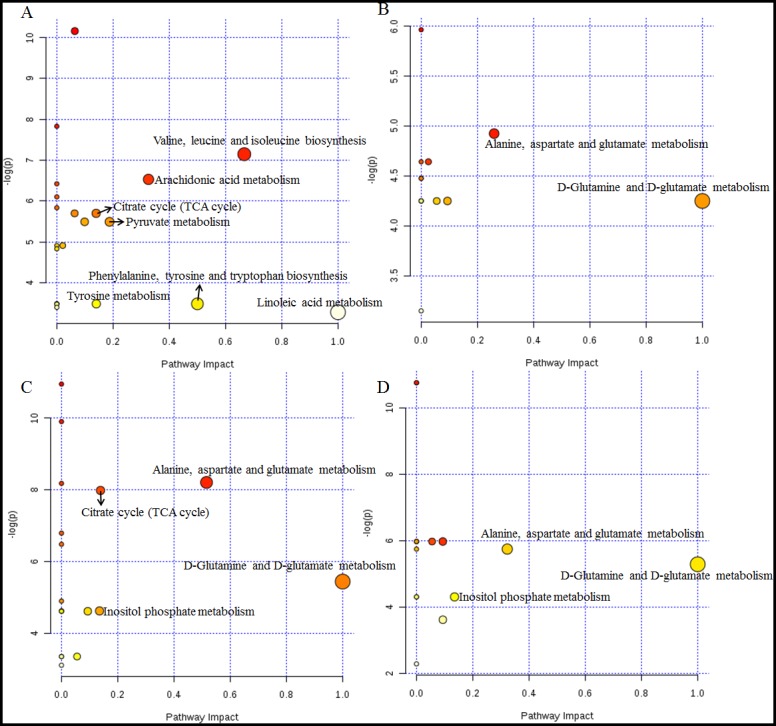
Metabolome view of the pathway analysis with Metaboanalyst 3.0 based on differential metabolites in Vehicle and XFZY groups The matched pathways are arranged by the -log( *p*) values of the pathway enrichment analysis on the Y-axis and the pathway impact values from the pathway topology analysis, which uses betweenness centrality measures to estimate node importance, on the X-axis. The colors of nodes are based on their *p*-values, and the radii are determined based on their pathway impact values. Small *p*-values and big pathway impact factors indicate that the pathway is highly influenced. **(A)** Representative disturbed metabolic pathways associated with TBI on day 1. **(B)** The most relevant metabolic pathways associated with effects of XFZY on day 1. **(C)** Representative disturbed metabolic pathways associated with TBI on day 3. **(D)** The most relevant metabolic pathways associated with effects of XFZY on day 3.

To identify the most relevant pathways, the threshold of impact value from the pathway analysis via MetaboAnalyst 3.0 is set at 0.10 [24]. Seven pathways (Figure 7A, Table 5) among the regulated pathways are filtered out as potential target pathways for TBI on day 1, four pathways (Figure 7C, Table 5) for TBI on day 3, two pathways for XFZY on day 1 (Figure 7B, Table 5), and three pathways for XFZY on day 3 (Figure 7D, Table 5).

**Table 5:** Result from metabolic pathway analysis with MetaboAnalyst 3.0

No.	Pathway Name	Total	Hits	-log(*p*)	Impact
TBI *vs*. Sham on day 1					
1	Valine, leucine and isoleucine biosynthesis	11	Valine, isoleucine, pyruvate	7.1454	0.66666
2	Arachidonic acid metabolism	36	Arachidonic acid	6.5286	0.32601
3	Citrate cycle (TCA cycle)	20	Oxoglutarate, pyruvate	5.6967	0.13983
4	Pyruvate metabolism	22	Lactic acid, pyruvate	5.4896	0.18754
5	Phenylalanine, tyrosine and tryptophan biosynthesis	4	Tyrosine	3.4817	0.5
6	Tyrosine metabolism	42	Tyrosine	3.4817	0.14045
7	Linoleic acid metabolism	5	Linoleic acid	3.2758	1
Vehicle *vs*. Sham on day 3					
1	Alanine, aspartate and glutamate metabolism	24	Aspartate, glutamate, succinate, oxoglutarate	8.2077	0.51582
2	Citrate cycle (TCA cycle)	20	Succinic acid, oxoglutaric acid, malic acid	7.9851	0.13885
3	D-Glutamine and D-glutamate metabolism	5	Glutamate, oxoglutarate	5.4504	1
4	Inositol phosphate metabolism	26	Inositol	4.6339	0.13525
XFZY *vs*. Vehicle on day 1					
1	Alanine, aspartate and glutamate metabolism	24	Glutamate, succinate	5.906	0.25949
2	D-Glutamine and D-glutamate metabolism	5	Glutamate	4.0325	1
XFZY *vs*. Vehicle on day 3					
1	Alanine, aspartate and glutamate metabolism	24	Glutamate, succinate, oxoglutarate	5.7528	0.32278
2	D-Glutamine and D-glutamate metabolism	5	Glutamate, oxoglutarate	5.2962	1
3	Inositol phosphate metabolism	26	Inositol	4.315	0.13525

Total is the total number of compounds in the pathway; the hits is the actually matched metabolites from the user uploaded data; the *p* is the *p*-value calculated from the enrichment analysis; the impact is the pathway impact value calculated from pathway topology analysis.

There are seven significant pathways out of a total of 21 pathways in rats post-TBI on day 1: valine, leucine and isoleucine biosynthesis, arachidonic acid metabolism, the citrate cycle (TCA cycle), pyruvate metabolism, phenylalanine, tyrosine and tryptophan biosynthesis, tyrosine metabolism, and linoleic acid metabolism. Related metabolite biomarkers of TBI disturbed by XFZY on day 1 are primarily involved in the following two significant pathways out of a total of 14 pathways: D-glutamine and D-glutamate metabolism, alanine, aspartate and glutamate metabolism.

**Figure 8:**
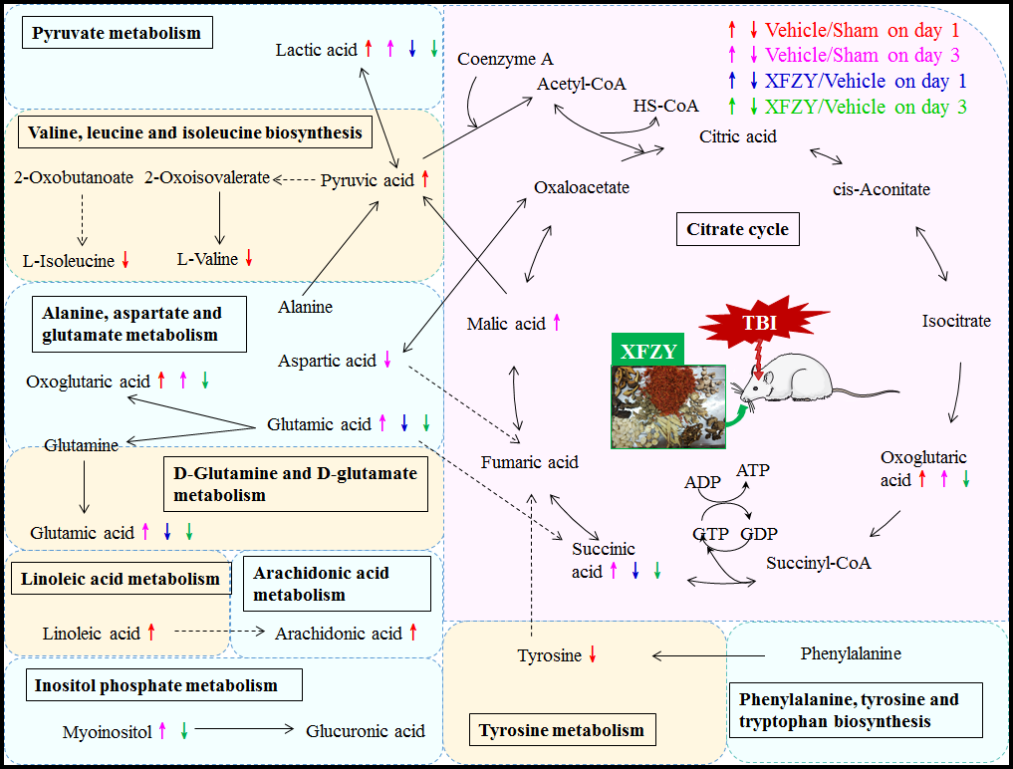
The most relevant metabolic pathways associated with TBI and XFZY on day 1 and day 3 Red and magenta represent significantly disturbed metabolites associated with TBI on day 1 and day 3, respectively; blue and green represent significant metabolites associated with XFZY treatment in TBI on day 1 and day 3, respectively. Up arrows represent increases, and down arrows represent decreases. Solid arrows indicate a single step reaction, and dotted arrows indicate multistep reactions.

There are four relevant pathways out of a total of 19 pathways in TBI on day 3: alanine, aspartate and glutamate metabolism, the citrate cycle, D-glutamine and D-glutamate metabolism, inositol phosphate metabolism. Among the four pathways, except the citrate cycle, the other three principal disturbed pathways are significantly associated with XFZY in rats with TBI on day 3, out of a total of 16 pathways. Metabolic pathway analysis revealed that differential metabolites are important for XFZY in TBI and are responsible for multiple pathways. By relating the metabolic pathways, a metabolic network of TBI-induced and XFZY-affected potential biomarkers was constructed (Figure 8).

## DISCUSSION

The present study describes a plasma metabolomics evaluation of acute TBI and the treatment of XFZY based on a GC-MS metabolomics approach combined with univariate and multivariate analyses. XFZY significantly ameliorates neurological impairment and lesion volume due to CCI on day 3. Furthermore, metabolomics analysis indicated that XFZY ameliorates the abnormal metabolic pattern of rats post-CCI on day 1 and day 3 by reversing changes of eight small-molecule metabolites (glutamic acid, lactic acid, oxoglutaric acid, succinic acid, ribitol, 3-hydroxybutyric acid, heptadecanoic acid, and myo-inositol) and three metabolic pathways (glutamine-glutamate metabolism, alanine-aspartate-glutamate metabolism, and inositol phosphate metabolism).

Our findings showed that metabolic changes of XFZY treatment on CCI are predominantly related to abnormal amino acid metabolism (glutamine-glutamate metabolism, alanine-aspartate-glutamate metabolism). Glutamic acid showed an increasing trend on day 1 and a significant increase on day 3 post-CCI, which is markedly decreased by XFZY at both time points, which is consisted with previous study [23]. The elevated glutamate, which can cause additional brain damage and influence clinical outcomes due to its excitotoxic and edema-aggravating potential [25]. It may mainly result from the impaired uptake of injured astrocytes and the ionic imbalances post-TBI since the glutamate transporter is a Na+- and K+-dependent antiporter [26]. XFZY decreased the concentration of glutamic acid in plasma potentially by regulating alanine-aspartate-glutamate metabolism and glutamine-glutamate metabolism, then restoring the uptake of glutamate and the ionic imbalances as well as increasing the activation of glutamic acid decarboxylase.

Changes post XFZY treatment are also mostly associated with regulation of carbohydrate metabolites (lactic acid, oxoglutaric acid, succinic acid, ribitol). Primary and secondary injuries of TBI induce transient increases in cerebral glucose utilization, rapidly followed by a prolonged depressed cerebral metabolism and elevated anaerobic glycolysis in tissue and extracellular lactate accumulation [27, 28]. Lactic acid, an end-product of glycolysis and traditionally portending a poor outcome [29], increases in rats post-CCI and decreases by XFZY on both day 1 and day 3. It shares a similar change with the previous study [23]. The accumulation of lactate to meet energy demand in TBI reflects tissue hypoxia, leukocyte accumulation at sites of infection or mitochondrial dysfunction by impeding pyruvate from entering the citrate cycle [30-32]. XFZY regulated the disorder of glycolysis, anaerobic and aerobic metabolism probably through increasing tissue oxygen delivery, inducing anti-inflammatory effects, and regulating mitochondrial dysfunction [16, 23].

Oxoglutaric acid and succinic acid are increased in rats post-CCI and decreased by XFZY treatment. They act as key intermediates in the citrate cycle, a vital metabolic pathway that consumes carbohydrates, amino acids and lipids to generate usable energy. Oxoglutaric acid could be synthesized through the action of oxidative deamination of glutamate in anaplerotic reaction, then could scavenge blood glutamate to increase the flow of glutamate from the brain into the blood, therefore, improve neurological outcomes after TBI in rats [33]. XFZY decreases the concentration of oxoglutaric acid in the plasma, and therefore, promotes anaplerotic reaction to decrease the level of glutamate. The accumulation of succinic acid may be produced from increased glutamine metabolism via oxoglutaric acid. Its accumulation can induce inflammation via stabilizing the transcription factor hypoxia-inducible factor-1α [34]. In addition, a study on ischemia reperfusion injury showed that under hypoxic conditions succinate is related to the abrupt and extensive production of mitochondrial reactive oxygen species, and then induce oxidative damage and the cellular apoptosis [35]. And the inhibition of ischemic succinate accumulation may ameliorate ischemia-reperfusion injury [36]. XFZY decreases the level of succinate, then partly reduces inflammation and mitochondrial reactive oxygen species.

Ribitol, one of the pentitols, significantly increased on day 1 after CCI and XFZY treatment. It acts as a precursor of tryptophan, then its increase indicated a reduction in tryptophan synthesis, which then suppresses inflammatory processes such as the proliferation of peripheral mononuclear cells, the activation of allogeneic immune cells and T-cell responses [37]. We hypothesize that the increase in ribitol in the plasma after TBI and XFZY may mediate the positive effects of lower tryptophan concentration and, thus, result in a slight inhibition of inflammatory responses.

Lipid mobilization is another distinctive trait of metabolic changes (3-hydroxybutyric acid, and heptadecanoic acid) after TBI and XFZY. A large increase of 3-hydroxybutyric acid in rats post-CCI and decrease by XFZY are noted. 3-hydroxybutyric acid, one of ketone bodies, can act as an alternative energy source from the breakdown of fatty acids via hepatic ketogenesis, and then be converted into acetyl-CoA to enter the citrate cycle [28, 38]. Heptadecanoic acid, an odd-chain saturated fatty acid, showed an increasing tendency post-TBI and a significant reduction by XFZY on day 1. It can be generated from the degradation of cerebrosides, sulfatides, and gangliosides of nervous tissue [39] and then generate propionyl-CoA to replenish the citrate cycle by succinyl-CoA. XFZY could decrease the alternative fuel demand, then decrease the level of heptadecanoic acid and 3-hydroxybutyric acid. Interestingly, in the present study, XFZY did not markedly decrease the level of arachidonic acid in the plasma as we previously reported in the brain tissue on day 1 and day 3 [16]. On day 1 post-CCI, the elevation of arachidonic acid was supported by previous studies on serum [40] and the injured brain [16] in rats post TBI. It indicated that XFZY preferentially decreases the level of arachidonic acid in the brain to that in the plasma, since lipids comprise the major portion of brain tissue accounting for almost half of the brain dry weight.

In addition to the three discussed metabolisms, another metabolite myo-inositol is an osmolyte primarily located in glia and absent in neurons [18]. It was reported to be elevated in putamen of adults with mild TBI [41], and occipital gray matter of pediatric TBI [42], with association with poor neurologic outcome. The increase is related to the reactive astrocytosis and microgliosis with increased glial content and proliferation after TBI [43]. Therefore, XFZY likely inhabited the reactive gliosis to reduce the myo-inositol concentration.

The significance of this work includes the following: (1) we identified 19 significantly disturbed metabolites in the plasma of rats post-CCI and following XFZY treatment. Among these, heptadecanoic acid and ribitol have not been previously associated with TBI or XFZY. Compared to a previous ^1^H-NMR study that revealed that XFZY increased the declined in N-acetyl aspartate, 4-hydroxybutyric acid, creatine and taurine and decreased the elevated lactic acid, glutamic acid, and choline [23], we also found a decrease effects of XFZY on lactic acid and glutamic acid. Besides, we first found XFZY increased the elevated ribitol, and decreased the elevated oxoglutaric acid, succinic acid, heptadecanoic acid, 3-hydroxybutyric acid and myo-inositol in rats post-CCI. (2) We also discovered three significantly perturbed metabolic pathways of XFZY on TBI through the pathway analysis and provide a network map to illustrate a new perspective of disturbed metabolites related to XFZY treatment on TBI.

Although we have identified altered metabolites, highly influenced pathways, and their possible role in TBI and XFZY intervention, our study still has several limitations. First, the metabolomics analysis was carried out only based on GC-MS data. Additional metabolomics methods or multiple metabolomics technologies (i.e., NMR, ultra-high performance liquid chromatography-tandem mass spectrometry, and spatial metabolomics) should be employed in future studies. Second, we only observed the metabolomics profile of plasma. Serum, urine, cerebrospinal fluid and brain samples should be evaluated in further studies to precisely reflect the pathological changes of TBI and therapeutic mechanisms of XFZY. Third, there is a limitation in the experimental grouping that we only set sham plus Vehicle group without sham plus XFZY group in this study. To design an herbal treatment without brain injury group to rule out any confounding factors from the treatment itself will be primarily considered in the future study. Finally, comprehensive analyses of the system pharmacology, transcriptomics, proteomics and metabolomics datasets could be performed in further studies to generate a system-wide view of the complex biological processes and the effects of XFZY. Although this study did not conclusively specify the regulation mechanism by which metabolites change and identify the upstream signaling pathway in XFZY-treated rats with TBI, we provide an initial description that the multiple changes in metabolites are important indicators and mechanisms behind its therapeutic effects.

## CONCLUSION

In this study, treatment with 9 g/kg XFZY for three consecutive days led to ameliorating neurological deficit and lesion volume in rats subjected to TBI. A GC-MS-based plasma metabolomics approach coupled with pattern recognition and pathway analysis uncovered that XFZY could reverse the abnormal metabolic profiles toward a normal state by interfering with eight different metabolites and three metabolic pathways. In summary, this study revealed the holistic metabolic changes of XFZY in rats with TBI and provided vital evidence for the efficacy of XFZY on TBI via multiple metabolites and metabolic pathways.

## MATERIALS AND METHODS

### Preparation of the XFZY Decoction extracts

All herbal medicines formulating the XFZY Decoction (namely, Semen *Persicae*, Flos *Carthami*, Radix *Angelicae Sinensis*, Radix *Rehmanniae*, Radix *Achyranthis Bidentatae*, Radix *Paeoniae Rubra*, Fructus *Aurantii*, Radix *Glycyrrhizae*, Rhizoma *Chuanxiong*, Radix *Platycodonis*, and Radix *Bupleuri*) were purchased from the Pharmacy of Xiangya Hospital of Central South University (Changsha, China), and authenticated by Prof. SY Hu, Department of Chinese Herbal Medicine of Central South University. Voucher specimens (Table 1) were deposited at the authors’ laboratory. The eleven herbs of XFZY were mixed in a ratio of 8:6:6:6:6:4:4:4:3:3:2 by weight to generate a freeze-dried powder of XFZY according to a previous report [44]. Finally, 1 g of the freeze-dried powder contained 4.9 g of crude herbs. The dried extract was dissolved in distilled water to 1 g crude drugs/ml (w/v) before use.

### Animals

Male Sprague-Dawley rats (n = 69) weighting 180-220 g were obtained from the Experimental Animal Center of Central South University. All experimental procedures were approved by the Ethical Committee for the Experimental Use of Animals at Central South University and were implemented according to the Guide for the Care and Use of Laboratory Animals. All rats were maintained in air-conditioned animal quarters under standard conditions (50 ± 10% relative humidity, 12/12 h light/dark cycle, 22 ± 2°C) and provided *ad libitum* water and food. All rats were acclimatized for at least 1 week and then fasted for 12 h before the experiment.

### Controlled cortical impact injury (CCI) model of TBI

Rats were anesthetized with 3% pentobarbital sodium (50 mg/kg) via intraperitoneal injection. CCI injury was performed using a PSI TBI-0310 Impactor (Precision Systems & Instrumentation LLC, Fairfax, VA, USA), according to a previous description [16]. Briefly, under the stereotaxic frame and sterile conditions, a midline longitudinal incision was made, and the skull was exposed. In addition, a left craniotomy was made using a portable drill and a 5 mm trephine over the left parietal cortex (center of the coordinates of the craniotomy relative to the bregma: 1 mm posterior, 1 mm lateral), and then the bone flap was removed. Rats were subjected to CCI using a pneumatic cylinder with a 3-mm flat-tip impounder, at a depth of 5.0 mm from the cortical surface, an impact velocity of 6.0 m/s, and a 500 ms impact duration. We used cyanoacrylate tissue glue to close the scalp and a built-in heating bed to maintain the body temperature of rats at 37°C throughout the operation. The rats in the Vehicle and XFZY groups were subjected to CCI, while those in the Sham group were only subjected to craniotomy.

### Experimental groups and administration of drugs

All rats were randomly divided into three groups in a blinded manner: Sham group (n=15), TBI+saline (Vehicle, n=27), TBI+XFZY (XFZY, n=27), and maintained in different cages. 24 rats of Vehicle and XFZY group were used for lesion volume assessment on day 1 and day 3. The Sham and Vehicle groups were administered with the same volume of normal saline after TBI by gavage, while the XFZY group was treated with the XFZY Decoction (9 g/kg) at 4 h after TBI and then every 24 h thereafter for 3 consecutive days.

### Assessments of neurological injury

The mNSS was used to assess posttraumatic neurological impairment, as previously described [16, 45]. The eighteen-point mNSS comprised motor tests (6 points), sensory tests (2 points), beam balance tests (6 points), reflexes absent, and abnormal movements (4 points). One point was given for failure of one task, and no points were given for a success. Thus, the severity of the injury is directly graded on a scale of 0 (normal) to 18 (maximal deficit). In each group, six rats were examined to evaluate the neurological function scores post-gavage 24 h on day 1 and 3. The tests were scored by two observers independently, who were blinded to the experimental design, and an average value was calculated for each rat on each day of testing.

### Lesion volume analysis

The lesion volume was determined by hematoxylin and eosin (H&E) staining, as described previously [44, 46]. After the completion of mNSS testing on day 1 and day 3 post-CCI, six rats from each group were anesthetized intraperitoneally with 10% chloral hydrate (40 mg/kg) and perfused transcardially with normal saline, followed by 4% paraformaldehyde. Brains were removed and stored in 4% paraformaldehyde for 1-2 days at room temperature, dehydrated with alcohols, and embedded in paraffin. Coronal brain sections (3 μm) at an interval of 90 μm and a total of about 10 sections of each brain were obtained on a rotary microtome and mounted on glass microscope slides. After drying at room temperature, the sections were deparaffinized in xylene, rehydrated, and stained with H&E. The images of the stained specimens were captured by a digital photo camera. And an observer blinded to experimental conditions using ImageJ analysis software (NIH) as per directions of the ImageJ developers (http://rsb.info.nih.gov/ij/) to estimate lesion size. The volume of the lesion region was calculated using the following formula:

$$\sum_{1}^{n}A_{j}\times d$$

where *A* is the corresponding lesion area, *n* is the total of sections and *d* is the distance between sections. The total lesion volume was calculated by numerical integration of all sections’ volume.

### Chemicals and reagents

BSTFA+1% TMCS (*N*, *O*-bis (trimethylsilyl) trifluoroacetamide with 1% trimethylchlorosilane), methoxyamine hydrochloride and pyridine, internal standard cis-10-nonadecenoic acid (C19:1, >99% purity), and the other 21 chemical standards of metabolites (shown in Table 2) were purchased from Sigma-Aldrich (Saint Louis, MO, USA). High-performance liquid chromatography grade methanol was purchased from Tedia Co., Inc. (Fairfield, USA).

### Sample collection and preparation

After assessing the functional score on day 1 and day 3, blood samples were collected into heparinized tubes via amputation of the end of the tails from 41 rats. The plasma was separated by centrifugation (6,000 rpm, 10 min, 4°C), and stored at -80°C until used in GC-MS analysis.

The process of plasma sample preparation was performed as previously described [47]. Briefly, each 100 μl aliquot of the plasma samples from all rats were protein precipitated with 300 μl ice-cold methanol, followed by the addition of 30 μl C19:1/methanol (1 mg/ml) as the internal standard. Then, the mixture was vortexed for 15 s and centrifuged for 10 min at 16,000 rpm and 4°C. The supernatant (330 μl) was carefully transferred into a 5-ml glass centrifugation tube and evaporated to dryness via gentle flow of nitrogen at room temperature. The residue was then subjected to a derivatization procedure involving 50 μl methoxyamine hydrochloride/pyridine (20 mg/ml) for incubation for 60 min at 70°C and then 100 μl BSTFA for incubation for another 60 min at 70°C. The final mixture was taken for GC-MS analysis in a random manner. In addition, 50 μl of each original sample were pooled to generate the quality control (QC), and aliquots of 100 μl of this pooled sample were taken through the same process. One QC sample was injected after every 6 sample injections to monitor the stability of GC-MS in this experiment.

### GC-MS analysis

GC-MS analysis of the derivatized samples was performed using a Shimadzu GC-2010 gas chromatography instrument and a Shimadzu QP2010 mass spectrometer (Shimadzu, Kyoto, Japan), equipped with an autosampler GL 221-34618. One microliter aliquots of the derivatized samples were injected in split mode (10:1) in an Agilent DB-5MS capillary column with a deactivated fused silica column (30 m×0.25 mm×0.25 μm; Agilent J & W Scientific, Folsom, CA, USA). High-purity helium was applied as the carrier gas at a constant flow rate of 1.0 ml/min. The column temperature was initially maintained at 70°C for 4 min, then increased to 300 °C at a rate of 8°C/min and held for 3 min. The septum purge was set at a constant flow rate of 3 ml/min. The temperatures of the ion source, interface and injector were 200°C, 250°C and 280°C respectively. MS detection was performed in electron impact mode (70 eV) in a full scan mode (m/z 35-800) with 0.2 s scan velocity, and the detector voltage was set to 0.96 kV.

### Data processing and statistical analysis

Raw spectrometric data including retention time (RT), chromatographic peak intensities, and the integrated mass spectra of each plasma sample were applied for the analysis. Metabolite identification of peaks-of-interest was performed by comparing the mass fragments with the NIST 05 mass spectral library using the NIST MS search software version 2.0 (the National Institute of Standards and Technology, Gaithersburg, MD, USA) and the characteristic ions. In addition, available commercial standards were used for confirmation. Only metabolic features with a relative standard deviation (RSD) for the relative peak areas of < 30% in QC samples were retained for the subsequent data analysis [48]. Thirty-seven metabolites were considered as the main endogenous metabolites, of which 21 metabolites were identified by their corresponding chemical standard substances. The peak areas of metabolites were normalized to the internal standard to obtain the semi-quantitative level of metabolites for further statistical analysis. The peak areas were extracted using our custom scripts to generate a data matrix, in which the rows represented the samples and the columns corresponded to the peak area ratios relative to the internal standard in the same chromatogram.

The resulting sample numbers (observations) and normalized peak area percent were introduced into the SIMCA-P 13.0 software package (Umetrics AB, Umeå, Sweden). For the multivariate analysis, the data were further mean-centered and scaled to unit variance for equal metabolite weighting and then evaluated by PCA to visualize the clustering trend, and to detect and exclude outlier datasets. After exclusion of sample outliers, PLS-DA and OPLS-DA were performed for supervised classification and discrimination between two groups. The results were presented in the form of score plots, in which each point represented an individual sample. The value of R2X and R2Y provide an estimate of how well the model fits the data and Q2 was used to describe the predictive ability of the class model [49]. The default seven-fold cross-validation of the OPLS-DA models was applied to avoid model overfitting, and 200 times permutation tests of the PLS-DA models were used to validate the reliability [50]. Then the VIP value plot, considering both the covariance p(corr) [1] and VIP [1] loading profiles resulting from the OPLS-DA model, was visualized the variable influence in a model. Thus, the features with VIP values greater than 1.0 were selected as metabolites of interest in the projection. The detected metabolites with differentiating ability were first tested for normality of the distribution with the Shapiro-Wilk test. If the distribution followed the normality assumption, two-tailed Student’s t-tests with Welch’s correction were subsequently applied; otherwise, Mann-Whitney U-tests were performed by SPSS 23.0 (International Business Machines Corp., Armonk, NY, USA). *p* < 0.05 was considered to indicate the significantly changed metabolites between two groups [51].

The data are reported as the means ± SD (n = 41), unless otherwise stated. mNSS was analyzed by two-way repeated-measures analysis of variance (RM ANOVA, group × time) followed by Holm-Sidak’s multiple comparisons tests, which were performed by Prism 6.0 (GraphPad). Significant metabolites were hierarchically clustered (average linkage) by Pearson correlation in heat maps, which were generated by MultiExperiment Viewer Version 4.9.0 (Mev, http://www.tm4.org/mev/). To identify the related metabolic pathways among the metabolites, all these differential identifications in the different groups of two time-points were simultaneously imported into MetaboAnalyst 3.0 (http://www.metaboanalyst.ca/) for further holistic pathway analysis. In this step, all compound names were matched into the KEGG IDs for the subsequent KEGG pathway analysis, and the Rattus norvegicus (rat) pathway library in the KEGG database was selected. “Global test” and “relative-betweenness centrality” were selected for calculating p-values of the pathway enrichment analysis and the impact values of the pathway topology analysis, respectively.

## SUPPLEMENTARY MATERIALS FIGURE


