# Supplementary material for: Metabolomics reveals the effect of Xuefu Zhuyu Decoction on plasma metabolism in rats with acute traumatic brain injury
Source: Oncotarget. 2017 Oct 16;8(55):94692–710. doi: 10.18632/oncotarget.21876 (PMC5706905; doi:10.18632/oncotarget.21876)
Supplement: Supplementary file 1 [file oncotarget-08-94692-s001.pdf]

Metabolomics reveals the effect of Xuefu Zhuyu Decoction on plasma metabolism in rats with acute traumatic brain injury

SUPPLEMENTARY MATERIALS

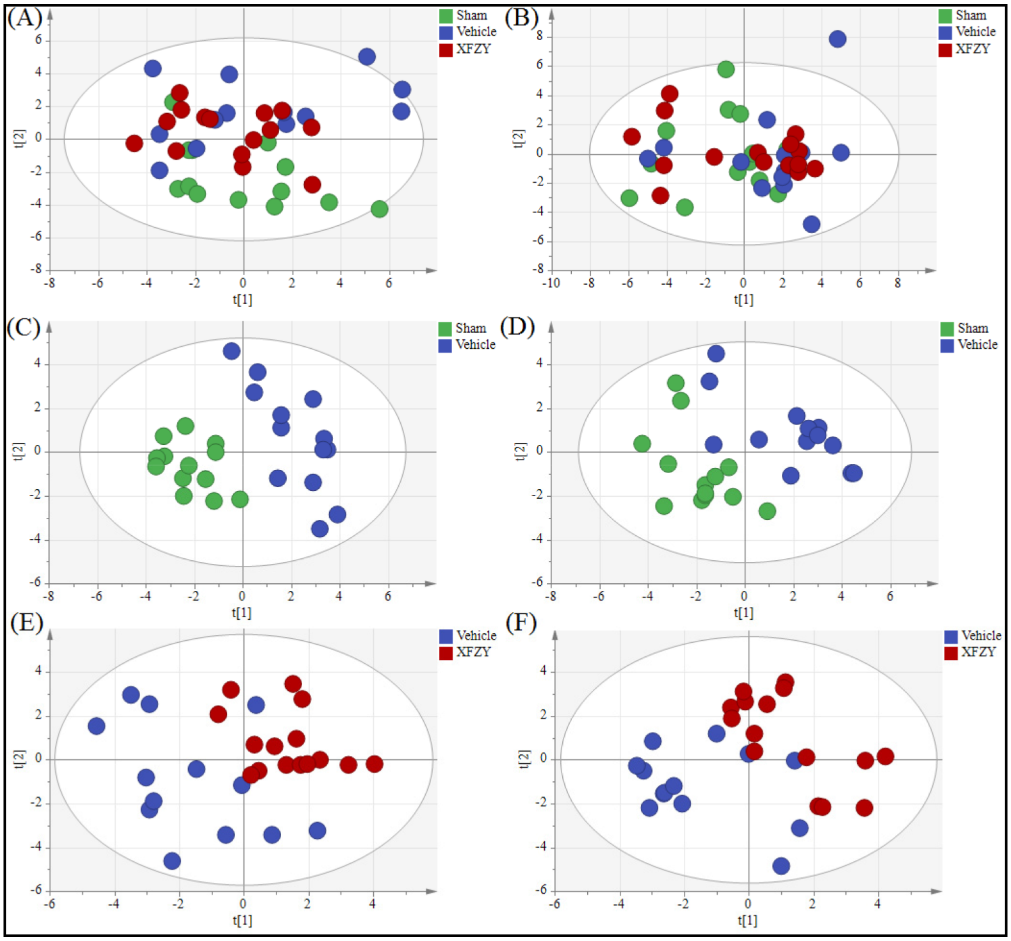

Supplementary Figure 1: Score plots from PCA and PLS-DA model between Sham, Vehicle, and XFZY groups. PCA score plot for the GC-MS analysis of plasma samples from the Sham group (green), the Vehicle group (dark blue) and the XFZY group (dark red) on day 1 **(A)** and day 3 **(B)**. PLS-DA scores plot of the Sham group (green) and the Vehicle group (dark blue) on day 1 **(C)** and day 3 **(D)**. PLS-DA scores plot of the Vehicle group (dark blue) and the XFZY group (dark red) on day 1 **(E)** and day 3 **(F)**.
